# Supplementary material for: Resolving the full spectrum of human genome variation using Linked-Reads
Source: Genome Res. 2019 Apr;29(4):635–45. doi: 10.1101/gr.234443.118 (PMC6442396; doi:10.1101/gr.234443.118)
Supplement: Supplemental Material [file supp_gr.234443.118_Supplemental_longranger-2.2.zip › longranger-2.2.tar.bz2!/lr2.2-public/lib/stan/cmdstan/stan/doc/api/html/filtered__values_8hpp_source.html]

Stan: src/stan/common/recorder/filtered\_values.hpp Source File


|  |  |
| --- | --- |
|  | Stan  2.5.0  probability, sampling & optimization |


- Main Page
- Related Pages
- Namespaces
- Classes
- Files

- File List
- File Members

All Classes Namespaces Files Functions Variables Typedefs Enumerations Enumerator Friends Macros Pages

- src
- stan
- common
- recorder

filtered\_values.hpp

Go to the documentation of this file.

1 #ifndef STAN\_\_COMMON\_\_RECORDER\_\_FILTERED\_VALUES\_HPP

2 #define STAN\_\_COMMON\_\_RECORDER\_\_FILTERED\_VALUES\_HPP

3

4 #include <stan/common/recorder/values.hpp>

5 #include <ostream>

6 #include <string>

7 #include <stdexcept>

8 #include <vector>

9

10 namespace stan {

11  namespace common {

12  namespace recorder {

13

14  template <class InternalVector>

15  class filtered\_values {

16  private:

17  size\_t N\_, M\_, N\_filter\_;

18  std::vector<size\_t> filter\_;

19  values<InternalVector> values\_;

20  std::vector<double> tmp;

21

22  public:

23

24  filtered\_values(const size\_t N,

25  const size\_t M,

26  const std::vector<size\_t>& filter)

27  : N\_(N), M\_(M), N\_filter\_(filter.size()), filter\_(filter),

28  values\_(N\_filter\_, M\_), tmp(N\_filter\_) {

29  for (size\_t n = 0; n < N\_filter\_; n++)

30  if (filter.at(n) >= N\_)

31  throw std::out\_of\_range("filter is looking for elements out of range");

32  }

33

34  filtered\_values(const size\_t N,

35  const std::vector<InternalVector>& x,

36  const std::vector<size\_t>& filter)

37  : N\_(N), M\_(0), filter\_(filter), N\_filter\_(filter.size()),

38  values\_(x), tmp(N\_filter\_) {

39  if (x.size() != filter.size())

40  throw std::length\_error("filter provided does not match dimensions of the storage");

41  if (N\_filter\_ > 0)

42  M\_ = x[0].size();

43  for (size\_t n = 0; n < N\_filter\_; n++)

44  if (filter.at(n) >= N\_)

45  throw std::out\_of\_range("filter is looking for elements out of range");

46  }

47

48  void operator()(const std::vector<std::string>& x) {

49  values\_(x);

50  }

51

52  template <class T>

53  void operator()(const std::vector<T>& x) {

54  if (x.size() != N\_)

55  throw std::length\_error("vector provided does not match the parameter length");

56  for (size\_t n = 0; n < N\_filter\_; n++)

57  tmp[n] = x[filter\_[n]];

58  values\_(tmp);

59  }

60

61  void operator()(const std::string x) {

62  values\_(x);

63  }

64

65  void operator()() {

66  values\_();

67  }

68

69  bool is\_recording() const {

70  return values\_.is\_recording();

71  }

72

73  const std::vector<InternalVector>& x() {

74  return values\_.x();

75  }

76  };

77

78  }

79  }

80 }

81

82 #endif

values.hpp

stan::common::recorder::filtered\_values::x

const std::vector< InternalVector > & x()

**Definition:** filtered\_values.hpp:73

stan::common::recorder::filtered\_values

**Definition:** filtered\_values.hpp:15

stan::common::recorder::values

**Definition:** values.hpp:14

stan::common::recorder::filtered\_values::is\_recording

bool is\_recording() const

**Definition:** filtered\_values.hpp:69

stan::common::recorder::filtered\_values::filtered\_values

filtered\_values(const size\_t N, const std::vector< InternalVector > &x, const std::vector< size\_t > &filter)

**Definition:** filtered\_values.hpp:34

stan::common::recorder::filtered\_values::operator()

void operator()(const std::vector< std::string > &x)

**Definition:** filtered\_values.hpp:48

stan::common::recorder::filtered\_values::operator()

void operator()()

**Definition:** filtered\_values.hpp:65

stan::math::size

int size(const std::vector< T > &x)

**Definition:** size.hpp:11

stan::common::recorder::filtered\_values::operator()

void operator()(const std::string x)

**Definition:** filtered\_values.hpp:61

stan::common::recorder::filtered\_values::operator()

void operator()(const std::vector< T > &x)

**Definition:** filtered\_values.hpp:53

stan::common::recorder::filtered\_values::filtered\_values

filtered\_values(const size\_t N, const size\_t M, const std::vector< size\_t > &filter)

**Definition:** filtered\_values.hpp:24


---

[ Stan Home Page ]

*© 2011–2014,
Stan Development Team.*
